# Supplementary material for: Longevity defined as top 10% survivors and beyond is transmitted as a quantitative genetic trait
Source: Nat Commun. 2019 Jan 7;10:35. doi: 10.1038/s41467-018-07925-0 (PMC6323124; doi:10.1038/s41467-018-07925-0)
Supplement: Supplementary file 1 — Supplementary Information [file 41467_2018_7925_MOESM1_ESM.pdf]

Supplementary table 1: Survival analysis for IPs by top 5% siblings and top 5% parents

	UPDB			LINKS		
	N (mean)	HR (95% CI)	p-value	N (mean)	HR (95% CI)	p-value
Top 5% parents (F1)						
0 (ref)	8149 (0.79)			8975 (0.88)		
1	1961 (0.19)	0.83 (0.79-0.88)	$8.19*10^{-11}$	1097 (0.11)	0.79 (0.74-0.84)	$2.07*10^{-12}$
2	136 (0.02)	0.70 (0.57-0.86)	$5.97*10^{-4}$	42 (0.01)	0.83 (0.60-1.16)	$2.75*10^{-1}$
Top 5% sibs (F2)						
0 (ref)	8169 (0.79)			9331 (0.92)		
1	1667 (0.17)	0.82 (0.74-0.91)	$1.48*10^{-4}$	712 (0.07)	0.79 (0.67-0.93)	$4.74*10^{-3}$
2+	410 (0.04)	0.75 (0.63-0.89)	$9.30*10^{-4}$	71 (0.01)	0.65 (0.42-1.02)	$6.12*10^{-2}$
LDS (F2)						
0 - non-religious(ref)	2753 (0.27)					
1 – baptized	512 (0.05)	0.69 (0.62-0.77)	$4.47*10^{-12}$	NA	NA	NA
2 - baptized + endowment	6736 (0.66)	0.82 (0.78-0.86)	$4.60*10^{-15}$	NA	NA	NA
3 - missing	245 (0.02)	0.87 (0.78-1.00)	$5.78*10^{-2}$	NA	NA	NA
Sibship size (F2)	10246 (6.28)	1.01 (1.00-1.02)	$1.04*10^{-1}$	10114 (6.34)	1.00 (0.99-1.01)	$4.35*10^{-1}$
Birth cohort, years (F2)	10246 (1868)	0.99 (>0.99<1.00)	$1.28*10^{-10}$	10114 (1835)	0.99 (>0.99<1.00)	$<1.00*10^{-15}$
Sex (F2)						
Man (ref)	5053 (0.49)			4776 (0.48)		
Women	5193 (0.51)	0.69 (0.65-0.74)	$<1.00*10^{-15}$	5338 (0.52)	1.01 (0.97-1.06)	$4.35*10^{-1}$
SES – OCC_1950 (F2)						
0 - High (ref)	315 (0.03)			67 (0.01)		
1	1482 (0.14)	1.12 (0.98-1.28)	$1.11*10^{-1}$	645 (0.06)	0.89 (0.69-1.15)	$3.64*10^{-1}$
2	400 (0.04)	1.14 (0.97-1.33)	$1.20*10^{-1}$	536 (0.05)	0.98 (0.76-1.27)	$8.66*10^{-1}$
3	352 (0.03)	1.22 (1.03-1.44)	$1.81*10^{-2}$	62 (0.01)	0.77 (0.54-1.10)	$1.51*10^{-1}$
4	187 (0.02)	1.10 (0.91-1.34)	$3.17*10^{-1}$	71 (0.01)	0.95 (0.67-1.34)	$7.67*10^{-1}$
5	891 (0.09)	1.23 (1.07-1.42)	$3.59*10^{-3}$	733 (0.07)	0.80 (0.62-1.03)	$8. 59*10^{-2}$
6	668 (0.07)	1.29 (1.12-1.49)	$5.11*10^{-4}$	311 (0.03)	0.86 (0.66-1.13)	$2.81*10^{-1}$
7	522 (0.05)	1.25 (1.07-1.45)	$4.92*10^{-3}$	759 (0.08)	0.82 (0.63-1.06)	$2.31*10^{-1}$
8	168 (0.02)	1.22 (0.99-1.50)	$5.94*10^{-2}$	574 (0.06)	0.85 (0.66-1.10)	$2.11*10^{-1}$
9 – Low	562 (0.05)	1.39 (1.19-1.61)	$2.11*10^{-5}$	3656 (0.36)	0.83 (0.65-1.07)	$1.47*10^{-1}$
999 - missing	4699 (0.46)	1.59 (1.40-1.81)	$1.74*10^{-12}$	2700 (0.26)	0.92 (0.72-1.18)	$4.93*10^{-1}$
Log likelihood	-69581			-77001		

Table corresponds to the CH curves in the top and bottom right panel of supplementary figure 2. Means represent a mean for a ontinuous variable and a proportion for a categorical variable. Additional covariates are: age mom at birth, birth order, birth intervals (in years), twin birth. When the p-value was lower than 1.00e-15 we indicated the P-value as <1.00e-15. LDS: the church of Jesus Christ of latter-day saints (Mormon church), SES: socio-economic status, OCC: occupational coding scheme of 1950. P-values are estimated with cox regression.

Supplementary table 2: Frailty survival analysis for Children of IPs by top 5% IP's and aunt and uncles of children

	UPDB			LINKS		
	N (mean)	HR (95% CI)	p-value	N (mean)	HR (95% CI)	p-value
Top 5% IP (F2)						
0 non LL (ref.)	54607 (0.90)			58196 (0.93)		
1 LL	6191 (0.10)	0.83 (0.80-0.86)	<1.00*10^{-15}	4278 (0.07)	0.83 (0.79-0.88)	5.66*10^{-13}
Top 5% aunts and uncles (F2)						
0 (ref.)	48154 (0.79)			57508 (0.92)		
1	10166 (0.17)	0.95 (0.92-0.98)	3.79*10^{-4}	4465 (0.07)	0.93 (0.89-0.97)	2.40*10^{-3}
2+	2478 (0.04)	0.91 (0.86-0.96)	1.60*10^{-3}	501 (0.01)	0.78 (0.68-0.90)	4.94*10^{-4}
Sibshipsize (F3)	60798 (8.89)	1.02 (1.01-1.02)	<1.00*10^{-15}	62474 (8.52)	1.00 (0.99-1.00)	5.92*10^{-1}
Birth year (F3)	60798 (1892)	0.99 (>0.99<1.00)	<1.00*10^{-15}	62474 (1867)	0.99 (>0.99<1.00)	6.52*10^{-12}
Sex (F3)						
Man (ref.)	31258 (0.51)			32136 (0.52)		
Women	29540 (0.49)	0.62 (0.60-0.63)	<1.00*10^{-15}	30338 (0.48)	0.64 (0.63-0.66)	<1.00*10^{-15}
Famid intercept (variance)	60798 (1.00)	0.34 (0.11)		62474 (1.00)	0.34 (0.11)	
BIC	60798 (1.00)	-23798.10		62474 (1.00)	-21555.34	

Additional covariates are: birth order, birth intervals (years), age mom at birth. Religion, Socio-economic status, twin birth have been stratified. When the p-value was lower than 1.00e-15 we indicated the P-value as <1.00e-15. BIC: Bayesian Information Criterion, Famid: family identifier. P-values are estimated with cox regression.

Supplementary table 3: Survival analysis for IP's by top 5% siblings for IPs without top 5% parents

	UPDB		
	non-longevous parents		
	N (mean)	HR (CI)	P-value
Top 5% sibs of RP			
0 (ref.)	6665 (0.82)		
1	1219 (0.15)	0.79 (0.70-0.90)	<0.0001
2+	165 (0.02)	0.77 (0.62-0.95)	0.0168

	LINKS		
	non-longevous parents		
	N (mean)	HR (CI)	P-value
Top 5% sibs of RP			
0 (ref.)	8354 (0.93)		
1	567 (0.06)	0.76 (0.63-0.92)	0.0045
2+	54 (0.01)	0.61 (0.37-0.99)	0.0500

Means represent a mean for a continuous variable and a proportion for a categorical variable. Additional covariates are: religion, sibship size, birth cohort, sex, socio-economic status, mother's age at birth, birth order, birth intervals, and twin birth. Here P-values were rounded to 4 digits. P-values are estimated with cox regression.

Supplementary table 4: frailty survival analysis for Children of IP's by top 5% aunt and uncles of children without top 5% parents

	UPDB		
	non-longevous RP + non-longevous spouse		
	N (mean)	HR (CI)	P-value
Top 5% aunts and uncles			
0 (ref.)	39338 (80)		
1	7934 (16)	0.93 (0.90-0.96)	<0.0001
2+	1816 (4)	0.91 (0.85-0.97)	0.0047
	LINKS		
	non-longevous RP + non-longevous spouse		
	N (mean)	HR (CI)	P-value
Top 5% aunts and uncles			
0 (ref.)	50165 (92)		
1	3737 (7)	0.93 (0.88-0.98)	0.0037
2+	345 (1)	0.73 (0.62-0.86)	<0.0001

Means represent a mean for a continuous variable and a proportion for a categorical variable. Additional covariates are: religion, sibship size, birth cohort, sex, socio-economic status, mother's age at birth, birth order, birth intervals, and twin birth. Here P-values were rounded to 4 digits. P-values are estimated with cox regression.

Supplementary table 5: Survival analysis for IP's by top 5% fathers and mothers

		UPDB			LINKS		
		N (mean)	HR (95% CI)	p-value	N (mean)	HR (95% CI)	p-value
F1-F2 (IP)	Top 5% parents						
	0 Both parents NL	7798 (0.79)			8927 (0.88)		
	1 Pa LL / Ma NL	1037 (0.10)	0.83 (0.77-0.89)	<0.0001	495 (0.5)	0.789(0.77-0.86)	<0.0001
	2 Ma LL / Pa NL	876 (0.9)	0.83 (0.78-0.89)	<0.0001	601 (0.6)	0.77 (0.70-0.84)	<0.0001
	3 Both parents LL	136 (0.2)	0.69 (0.58-0.82)	<0.0001	42 (0.1)	0.86 (0.63-1.16)	0.3368
F2-F3 (full)	Top 5% parents						
	0 Both parents NL	47960 (0.80)			53422 (0.86)		
	1 Pa LL / Ma NL	6176 (0.10)	0.86 (0.83-0.89)	<0.0001	4668 (0.07)	0.85 (0.81-0.89)	<0.0001
	2 Ma LL / Pa NL	5352 (0.09)	0.83 (0.79-0.86)	<0.0001	3783 (0.06)	0.82 (0.78-0.86)	<0.0001
	3 Both parents LL	754 (0.01)	0.68 (0.61-0.76)	<0.0001	438 (0.01)	0.63 (0.54-0.73)	<0.0001

Means represent a mean for a continuous variable and a proportion for a categorical variable. Additional covariates are: religion, sibship size, birth cohort, sex, socio-economic status, mother's age at birth, birth order, birth intervals, and twin birth. PA=father, MA=mother, NL=non-longevous, LL=longevous, NL=non-longevous, longevous was defined as belonging to the top 10% of a persons' birth cohort. Here P-values
were rounded to 4 digits. P-values are estimated with cox regression.

Supplementary table 6 - construction of final statistical models UPDB - 10 percent

	Model 1		Model 2		Model 3		Model 4	
	N (mean)	HR (95% CI)	N (mean)	HR (95% CI)	N (mean)	HR (95% CI)	N (mean)	HR (95% CI)
Top 10% parents (F1)								
0 (ref)	6640 (0.65)				7861 (0.78)		6640 (0.65)	
1	3167 (0.31)	0.89 (0.84-0.93)			2096 (0.20)	0.90 (0.86-0.95)	3167 (0.31)	0.88 (0.83-0.92)
2	439 (0.4)	0.74 (0.66-0.83)			184 (0.2)	0.76 (0.67-0.85)	439 (0.4)	0.73 (0.65-0.83)
Top 10% sibs (F2)								
0 (ref)			6720 (0.66)		8644 (0.85)		6720 (0.66)	
1			2495 (0.24)	0.82 (0.75-0.89)	1256 (0.13)	0.83 (0.76-0.90)	2495 (0.24)	0.82 (0.76-0.90)
2+			1031 (0.10)	0.74 (0.66-0.82)	214 (0.2)	0.76 (0.68-0.84)	1031 (0.10)	0.74 (0.66-0.82)
LDS (F2)								
0 - non-religious(ref)							2753 (0.27)	
1 – baptized							512 (0.05)	NA
2 - baptized + endowment							6736 (0.66)	NA
3 - missing							245 (0.02)	NA
Sibship size (F2)							10246 (6.28)	1.01 (1.00-1.02)
Birth cohort, years (F2)							10246 (1868)	0.99 (>0.99<1.00)
Sex (F2)								
Man (ref)							5053 (0.49)	
Women							5193 (0.51)	0.71 (0.67-0.76)
SES – OCC_1950 (F2)								
0 - High (ref)							315 (0.03)	
1							1482 (0.14)	1.16 (1.01-1.34)
2							400 (0.04)	1.19 (1.00-1.40)
3							352 (0.03)	1.24 (1.05-1.48)
4							187 (0.02)	1.14 (0.93-1.40)
5							891 (0.09)	1.31 (1.13-1.52)
6							668 (0.07)	1.34 (1.15-1.56)
7							522 (0.05)	1.27 (1.08-1.50)
8							168 (0.02)	1.21 (0.97-1.50)
9 – Low							562 (0.05)	1.48 (1.26-1.73)
999 - missing							4699 (0.46)	1.61 (1.40-1.84)

-Means represent a mean for a continuous variable and a proportion for a categorical variable. Additional covariates are: age mom at birth, birth order, birth intervals (in years), and twin birth. LDS: the church of Jesus Christ of latter-day saints (Mormon church), SES: socio-economic status, OCC: occupational coding scheme of 1950. P-values are estimated with cox regression.

Supplementary table 7 - construction of final statistical models LINKS - 10 percent

	Model 1		Model 2		Model 3		Model 4	
	N (mean)	HR (95% CI)	N (mean)	HR (95% CI)	N (mean)	HR (95% CI)	N (mean)	HR (95% CI)
Top 10% parents (F1)								
0 (ref)	7861 (0.78)				7861 (0.78)		7861 (0.78)	
1	2096 (0.20)	0.81 (0.77-0.86)			2096 (0.20)	0.82 (0.78-0.86)	2096 (0.20)	0.82 (0.78-0.86)
2	184 (0.2)	0.68 (0.58-0.81)			184 (0.2)	0.69 (0.58-0.82)	184 (0.2)	0.69 (0.58-0.82)
Top 10% sibs (F2)								
0 (ref)			8644 (0.85)		8644 (0.85)		8644 (0.85)	
1			1256 (0.13)	0.82 (0.72-0.92)	1256 (0.13)	0.83 (0.73-0.94)	1256 (0.13)	0.82 (0.73-0.93)
2+			214 (0.2)	0.72 (0.56-0.92)	214 (0.2)	0.76 (0.59-0.97)	214 (0.2)	0.75 (0.58-0.96)
LDS (F2)								
0 - non-religious(ref)								
1 – baptized							NA	NA
2 - baptized + endowment							NA	NA
3 - missing							NA	NA
Sibship size (F2)							10114 (6.34)	1.01 (1.00-1.02)
Birth cohort, years (F2)							10114 (1835)	0.99 (>0.99<1.00)
Sex (F2)								
Man (ref)							4776 (0.48)	
Women							5338 (0.52)	1.01 (0.96-1.06)
SES – OCC_1950 (F2)								
0 - High (ref)							67 (0.01)	
1							645 (0.06)	0.88 (0.68-1.14)
2							536 (0.05)	0.97 (0.75-1.27)
3							62 (0.01)	0.76 (0.53-1.10)
4							71 (0.01)	0.99 (0.70-1.40)
5							733 (0.07)	0.80 (0.62-1.04)
6							311 (0.03)	0.86 (0.65-1.13)
7							759 (0.08)	0.84 (0.65-1.10)
8							574 (0.06)	0.85 (0.65-1.11)
9 – Low							3656 (0.36)	0.83 (0.65-1.07)
999 - missing							2700 (0.26)	0.93 (0.72-1.20)

Means represent a mean for a continuous variable and a proportion for a categorical variable. Additional covariates are: age mom at birth, birth order, birth intervals (in years), twin birth. LDS: the church of Jesus Christ of latter-day saints (Mormon church), SES: socio-economic status, OCC: occupational coding scheme of 1950. P-values are estimated with cox regression.

Supplementary table 8: Survival analysis for IP's by top 10% siblings among IPs without top 10% parents

	UPDB		
	non-longevous parents		
	N (mean)	HR (CI)	P-value
Top 10% sibs of RP			
0 (ref.)	4639 (0.70)		
1	1473 (0.22)	0.85 (0.79-0.91)	<0.0001
2+	528 (0.8)	0.78 (0.67-0.90)	<0.0001
	LINKS		
	non-longevous parents		
	N (mean)	HR (CI)	P-value
Top 10% sibs of RP			
0 (ref.)	6867 (0.87)		
1	886 (0.11)	0.78 (0.72-0.85)	<0.0001
2+	108 (0.2)	0.72 (0.53-0.99)	0.0429

-Means represent a mean for a continuous variable and a proportion for a categorical variable. Additional covariates are: religion, sibship size, birth cohort, sex, socio-economic status, mother's age at birth, birth order, birth intervals, and twin birth. Here P-values were rounded to 4 digits. P-values are estimated with cox regression.

Supplementary table 9: Survival analysis for IP's by top 10% fathers and mothers

		UPDB			LINKS		
		N (mean)	HR (95% CI)	p-value	N (mean)	HR (95% CI)	p-value
F1-F2 (IP)	Top 10% parents						
	0 Both parents NL	6334 (0.64)			7817 (0.77)		
	1 Pa LL / Ma NL	1653 (0.17)	0.88 (0.84-0.93)	<0.0001	1124 (0.11)	0.83 (0.78-0.89)	<0.0001
	2 Ma LL / Pa NL	1421 (0.15)	0.84 (0.79-0.89)	<0.0001	940 (0.09)	0.80 (0.75-0.86)	<0.0001
	3 Both parents LL	439 (0.4)	0.73 (0.66-0.80)	<0.0001	184 (0.03)	0.72 (0.62-0.83)	<0.0001
F2-F3 (full)	Top 10% parents						
	0 Both parents NL	38423 (0.64)			45644 (0.73)		
	1 Pa LL / Ma NL	10522 (0.17)	0.89 (0.86-0.92)	<0.0001	8643 (0.14)	0.86 (0.83-0.89)	<0.0001
	2 Ma LL / Pa NL	8969 (0.15)	0.86 (0.83-0.89)	<0.0001	6360 (0.10)	0.82 (0.79-0.86)	<0.0001
	3 Both parents LL	2328 (0.4)	0.72 (0.68-0.76)	<0.0001	1664 (0.03)	0.73 (0.67-0.79)	<0.0001

Means represent a mean for a continuous variable and a proportion for a categorical variable. Additional covariates are: religion, sibship size, birth cohort, sex, socio-economic status, mother's age at birth, birth order, birth intervals, and twin birth. PA=father, MA=mother, NL=non-longevous, LL=longevous, NL=non-longevous, longevous was defined as belonging to the top 10% of a persons' birth cohort. Here P-values were rounded to 4 digits. P-values are estimated with cox regression.

Supplementary table 10: Sex specific survival analysis for IP's by top 10% parents

		UPDB			LINKS		
		N (mean)	HR (95% CI)	p-value	N (mean)	HR (95% CI)	p-value
F1-F2 (IP)	Top 10% parents (F1)						
	0	6640 (0.65)			7861 (0.78)		
	1	3167 (0.31)	0.85 (0.75-0.96)	0.0067	2096 (0.20)	0.83 (0.78-0.91)	<0.0001
	2	439 (0.4)	0.68 (0.59-0.80)	<0.0001	184 (0.2)	0.61 (0.48-0.76)	<0.0001
	Sex						
	Man (ref)	5053 (0.49)			4776 (0.48)		
	Women	5193 (0.51)	0.73 (0.68-0.78)	<0.0001	5338 (0.52)	1.02 (0.97-1.08)	0.3600
	Sex* Top 10% parents (F1)						
	0 Top 10 parents | women (ref)	6640 (0.65)			7861 (0.78)		
	1 Top 10 parents | women	3167 (0.31)	0.92 (0.78-1.10)	0.3440	2096 (0.20)	0.96 (0.86-1.10)	0.4041
	2 Top 10 parents | women	439 (0.4)	1.10 (0.89-1.35)	0.4010	184 (0.2)	1.36 (0.97-1.91)	0.0748
F2-F3 (Child of IP - one LL parent)	Top 10% parents (F1)						
	0	48619 (0.80)			53378 (0.85)		
	1	12179 (0.20)	0.83 (0.77-0.89)	<0.0001	9096 (0.15)	0.86 (0.78-0.94)	<0.0017
	Sex						
	Man (ref)	31258 (0.51)			32136 (0.52)		
	Women	29540 (0.49)	0.56 (0.55-0.58)	<0.0001	30338 (0.48)	0.64 (0.63-0.66)	<0.0001
	Sex* Top 10% parents (F1)						
	0 Top 10 parents | women (ref)	48619 (0.80)			53378 (0.85)		
	1 Top 10 parents | women	12179 (0.20)	1.03 (0.98-1.08)	0.2390	9096 (0.15)	1.00 (0.94-1.05)	0.1863
F2-F3 (Child of IP - two LL parents)	Top 10% parents (F1)						
	0	58672 (0.96)			60962 (0.97)		
	2	2126 (0.04)	0.68 (0.57-0.80)	<0.0001	1512 (0.03)	0.78 (0.63-0.96)	0.0138
	Sex						
	Man (ref)	31258 (0.51)			32136 (0.52)		
	Women	29540 (0.49)	0.56 (0.55-0.58)	<0.0001	30338 (0.48)	0.64 (0.63-0.66)	<0.0001
	Sex* Top 10% parents (F1)						
	0 Top 10 parents | women (ref)	58672 (0.96)			60962 (0.97)		
	2 Top 10 parents | women	2126 (0.04)	1.09 (0.99-1.21)	0.0812	1512 (0.03)	1.00 (0.87-1.13)	0.1947

Means represent a mean for a continuous variable and a proportion for a categorical variable. Additional covariates are: religion, sibship size, birth cohort, sex, socio-economic status, mother's age at birth, birth order, birth intervals, and twin birth. PA=father, MA=mother, NL=non-longevous, LL=longevous, NL=non-longevous, longevous was defined as belonging to the top 10% of a persons' birth cohort. Here P-values were rounded to 4 digits. P-values are estimated with cox regression.

Supplementary table 11: frailty survival analysis for Children of IP’s By top 10% aunt and uncles of children without top 10% parents

	UPDB		
	non-longevous RP + non-longevous spouse		
	N (mean)	HR (CI)	P-value
Top 10% aunts and uncles			
0 (ref.)	26475 (0.67)		
1	9374 (0.24)	0.96 (0.93-0.99)	0.0170
2+	3516 (0.8)	0.90 (0.86-0.95)	<0.0001

	LINKS		
	non-longevous RP + non-longevous spouse		
	N (mean)	HR (CI)	P-value
Top 10% aunts and uncles			
0 (ref.)	40031 (0.86)		
1	5651 (0.12)	0.95 (0.91-0.99)	0.0134
2+	890 (0.2)	0.81 (0.73-0.90)	<0.0001

-Means represent a mean for a continuous variable and a proportion for a categorical variable. Additional covariates are: religion, sibship size, birth cohort, sex, socio-economic status, mother’s age at birth, birth order, birth intervals, and twin birth. Here P-values were rounded to 4 digits. P-values are estimated with cox regression.

Supplementary table 12: Selection criteria for the random sampling of F2 IPs

Selection	Motivation
-At least 1 identifiable child	Part of the study focused on children, hence children needed to be available for the index persons
-At least 1 identifiable sibling	To ensure that the influence of siblings could be analyzed we excluded families with only a single child. In addition this was a method to make sure that dummy families were excluded
-A spouse should be identified	Part of the study focused on spouses who married to longevous index persons. For this, and because index persons needed to have a child, a spouse was required to be available
-A known sex	To be able to distinguish between males and females, the sex of at least the index persons needed to be available
-Availability of a birth date<br>-Availability of a death	To be able to study the survival of the complete group of index persons with the best possible data, a date of birth and a date of death needed to be available. In addition, in the LINKS data selecting on an available birth and death date was a quality check that made sure that index person was indeed part of the identified family.
-In the UPDB: should be identified on a genealogy record	All genealogy records are verified. Hence, this was a double check to make sure that the index person was indeed part of the identified family.

UPDB: Utah Population Database, LINKS: LINKing System for historical family reconstruction.

Supplementary table 13: Demographic spread for included UPDB persons

**Role**	**Fathers**	**Mothers**	**IPs**	**Siblings**	**Spouses**	**Children**
Lived in Utah (%)	7600 (80)	8478 (87)	9901 (97)	32026 (70)	9998 (97)	49839 (92)
Not lived in Utah (%)	1884 (20)	1229 (13)	345 (3)	13674 (30)	356 (3)	4236 (8)
**Birth continent**	**America**	**Europe**	**Asia**	**Africa**	**Australia**	**Other**
Lived in Utah	85141 (91)	11490 (73)	35 (65)	4 (100)	195 (73)	171 (34)
Not lived in Utah	8310 (9)	4363 (27)	19 (45)	0 (0)	73 (27)	329 (66)

Numbers are based on uncensored individuals. Numbers are based on the UPDB only.

Supplementary figure 1: Histogram of IP age at death and IP survival percentiles

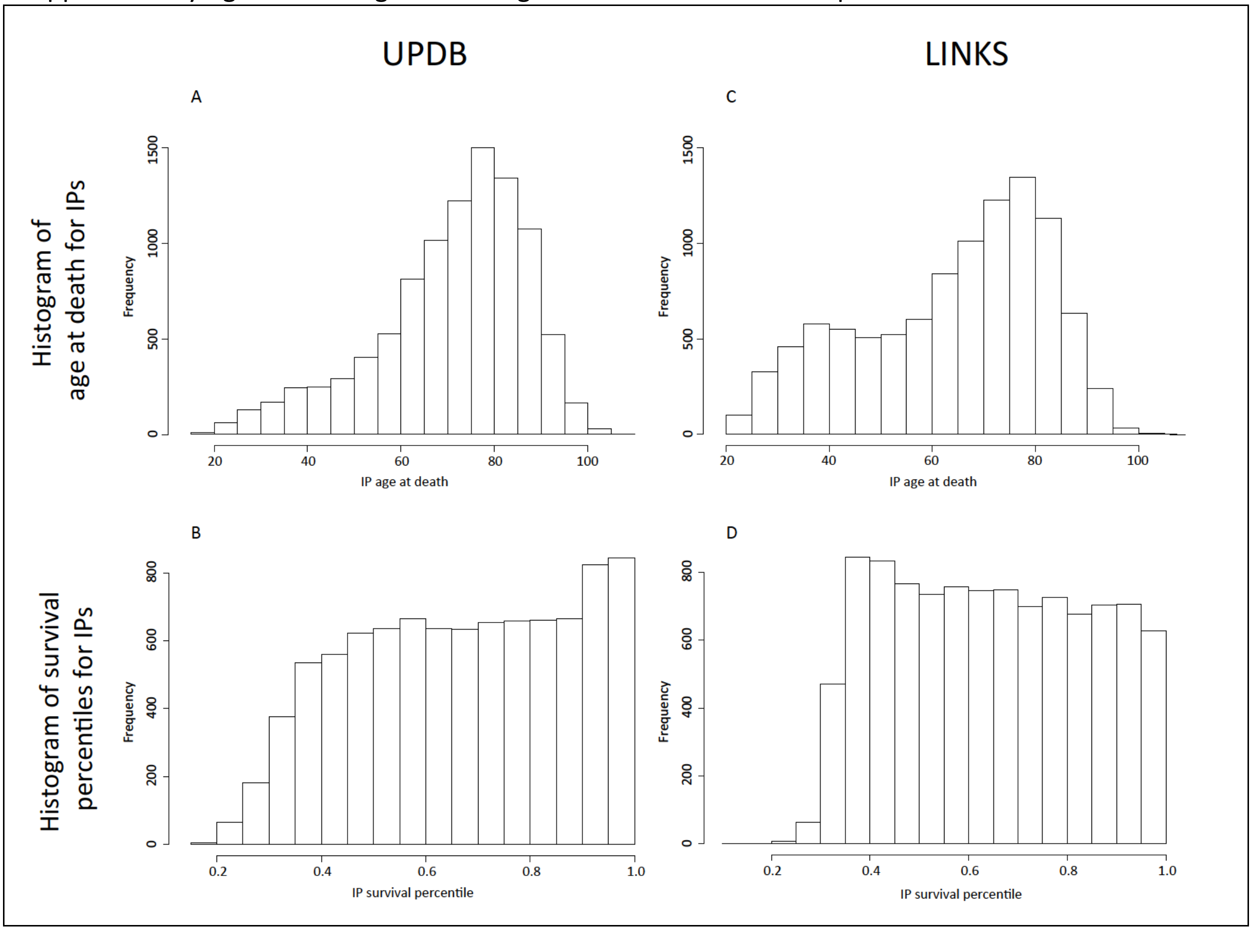

Panel A and C depict a histogram for the IPs ages at death with the UPDB and LINKS data respectively. Panel B and D depict a histogram for the IPs survival percentiles with the UPDB and LINKS data respectively. IP=Index Person.

Supplementary figure 2: Median age at death and survival percentile for IPs grouped by their parental and sibling survival in mutual exclusive groups

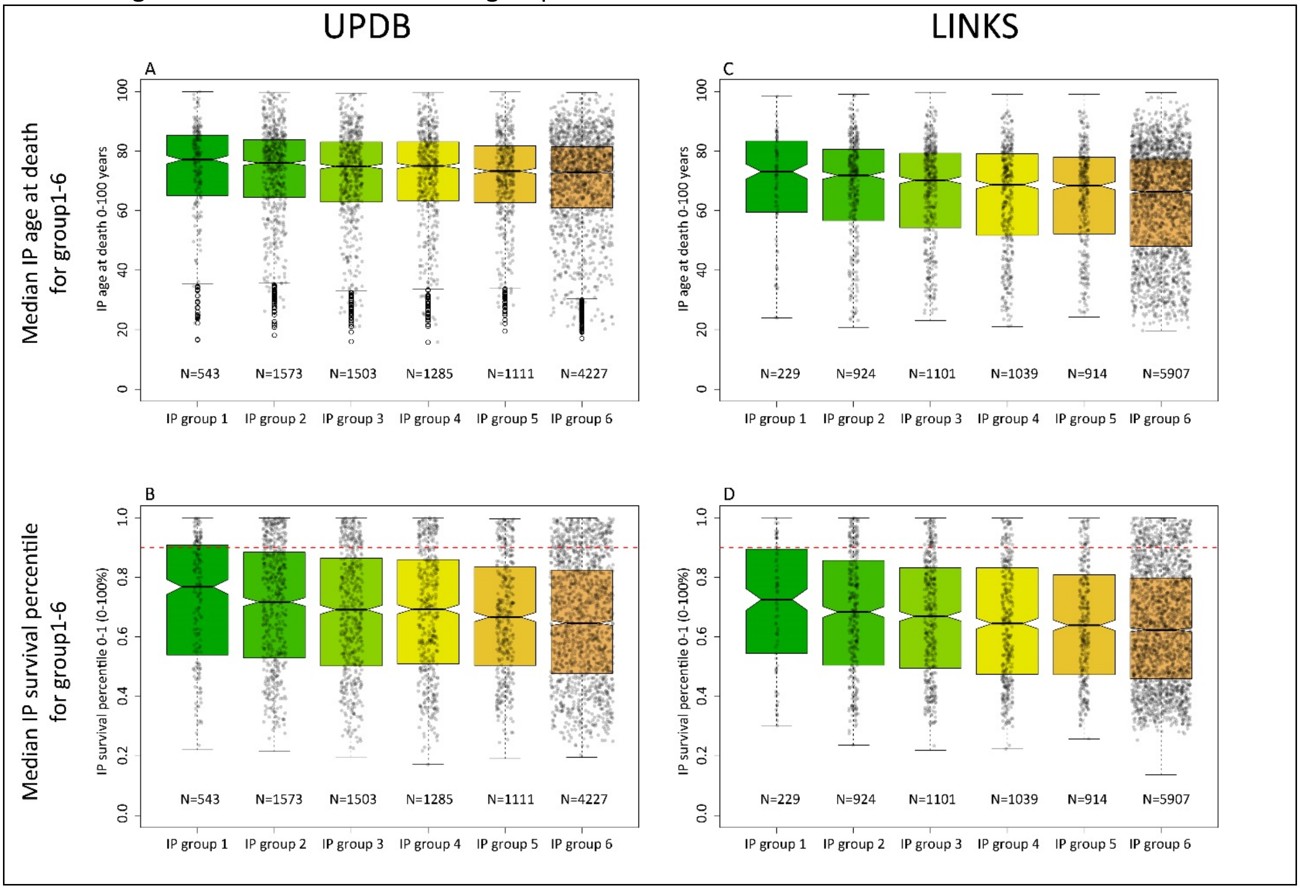

This figure relates to main figure 3 and shows the median + quantiles and variation for IPs' age at death on the top row (panel A and C). The bottom row (panel B and D) shows the median + quantiles and variation for IPs' survival percentiles. Nodes are based on 1/6th of the total sample size for illustrative purposes. The red lines on the bottom row represent the cut-off for the top 10 percent surviving IPs for the different groups. Similar to the decrease in HR for the different groups illustrated in main figure 2 and the increase in age at death or survival percentile for the different groups illustrated in this figure, there is an increase in top 10% surviving IPs. For the UPDB data 17% of the total number of IPs in group 6 belongs to the top 10% survivors, this is 19% for group 5, 23% for group 4, 26% for group 3, 29% for group 2, and 37% for group 1. For the LINKS data the numbers, in similar order, are 13%, 14%, 16%, 18%, 23% and 32%.

Supplementary figure 3: Survival of IPs with parents and siblings belonging to the 1st until 60th percentile survivors of their birth cohort

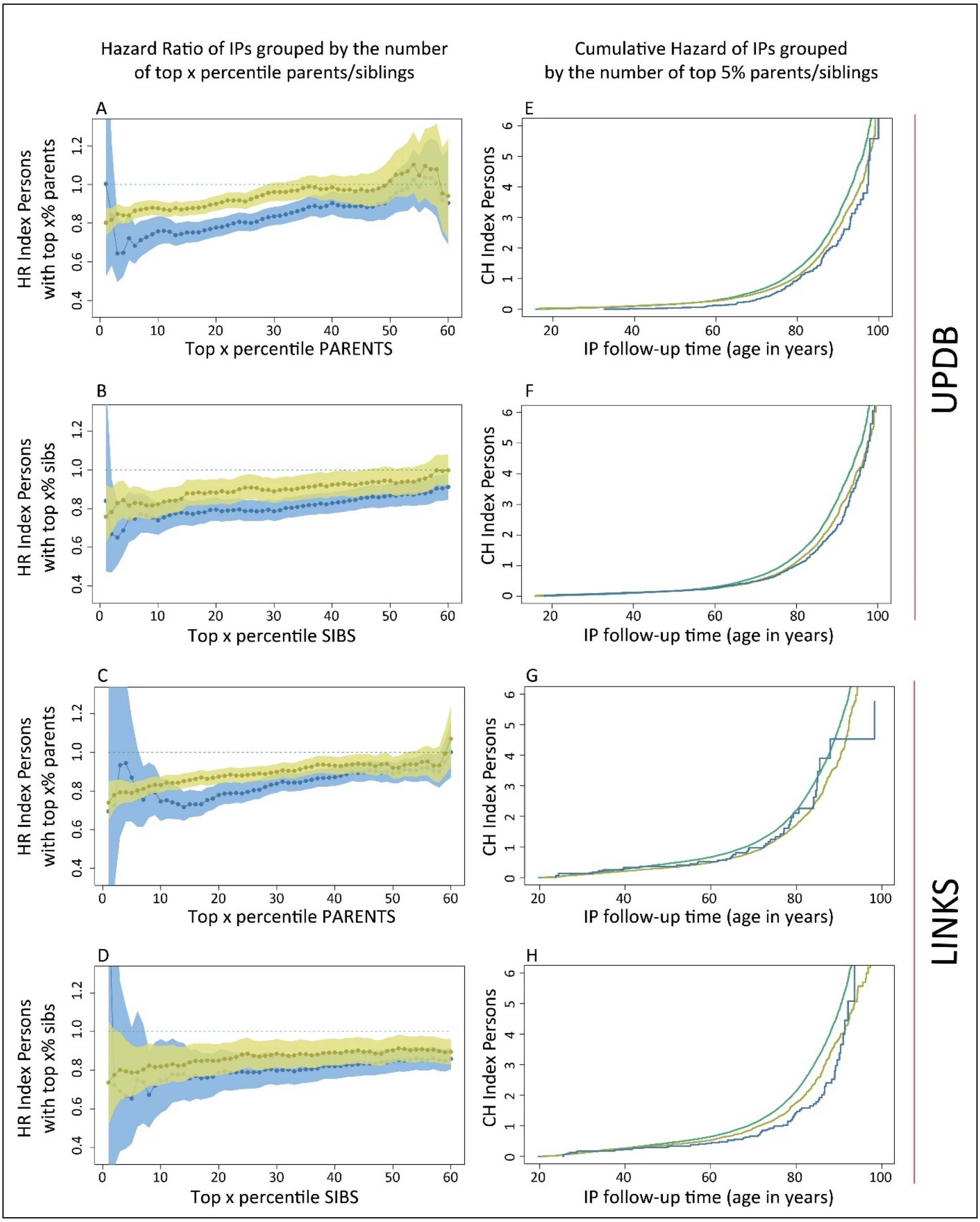

This figure depicts the Hazard Ratio (HR) for IPs (left column, panel A-D) with 1 and 2 parents or 1 and 2+ siblings belonging to the top x percentile (x = 1,2,3, …, 60) of survivors of their birth cohort. The percentile groups (x-axis) are mutually inclusive, meaning that a first-degree family member who belonged to the top 1% also belonged to the top 5% etc. The figure also depicts the Cumulative Hazard (CH) for index persons (IPs, right column, panel E-H) with 1 and 2 parents or 1 and 2+ siblings who belong to the top 5%. Green (dotted) lines present the reference group of 0 top x percentile parents or siblings, yellow lines represent 1 top x percentile parents or siblings, blue lines represent 2 or 2+ top x percentile siblings. Left column: x-axes represent the top x birth cohort based survival percentile, the y-axes represent the hazard ratio (HR) of dying for IPs having 1 and 2 or 2+ top x percentile parents or siblings compared to having 0 top x percentile parents or siblings. Right column: x-axes represent IP years of survival, y-axes represent the IPs' cumulative hazard of dying while having 1 and 2 or 2+ top 5th percentile parents or siblings compared to having 0 top 5th percentile parents or siblings. All estimates are adjusted for religion (UPDB only), sibship size, birth cohort, sex, socio-economic status, mother's age at birth, birth order, birth intervals, twin birth, and number of top 5% parents or number of top 5% siblings for the sibling and parent analyses respectively. Error bars represent confidence intervals.

Supplementary figure 4: Kaplan-Meier and Nelson-Aalen plots for IPs by the longevity of their parents and siblings at the top 5%

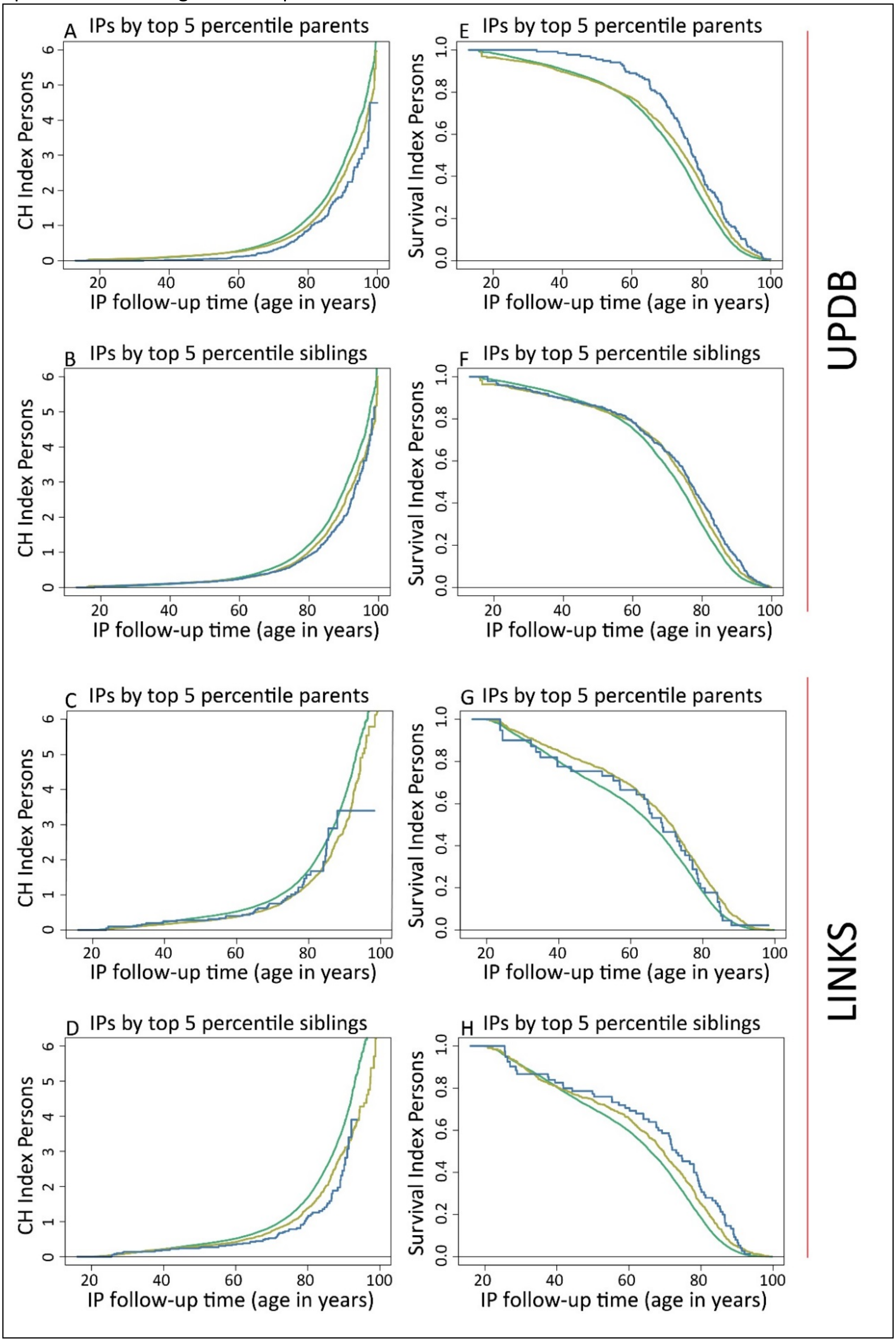

Green lines present 0 top x percentile parents or siblings, yellow lines represent 1 top x percentile parents or siblings, blue lines represent 2 or 2+ longevous parents or siblings. CH=Cumulative Hazard, IP=Index Person. Left column depicts the Kaplan-Meier curves, right column depicts the Nelson-Aalen curves. Panel A, B, E, and F represent UPDB IPs, panel C, D, G, and H represent LINKS IPs. CH=Cumulative Hazard, IP=Index Person.

Supplementary figure 5: Nelson-Aalen plots for children of IPs by the longevity of their parents (IPs and spouses of IPs) at the top 5%

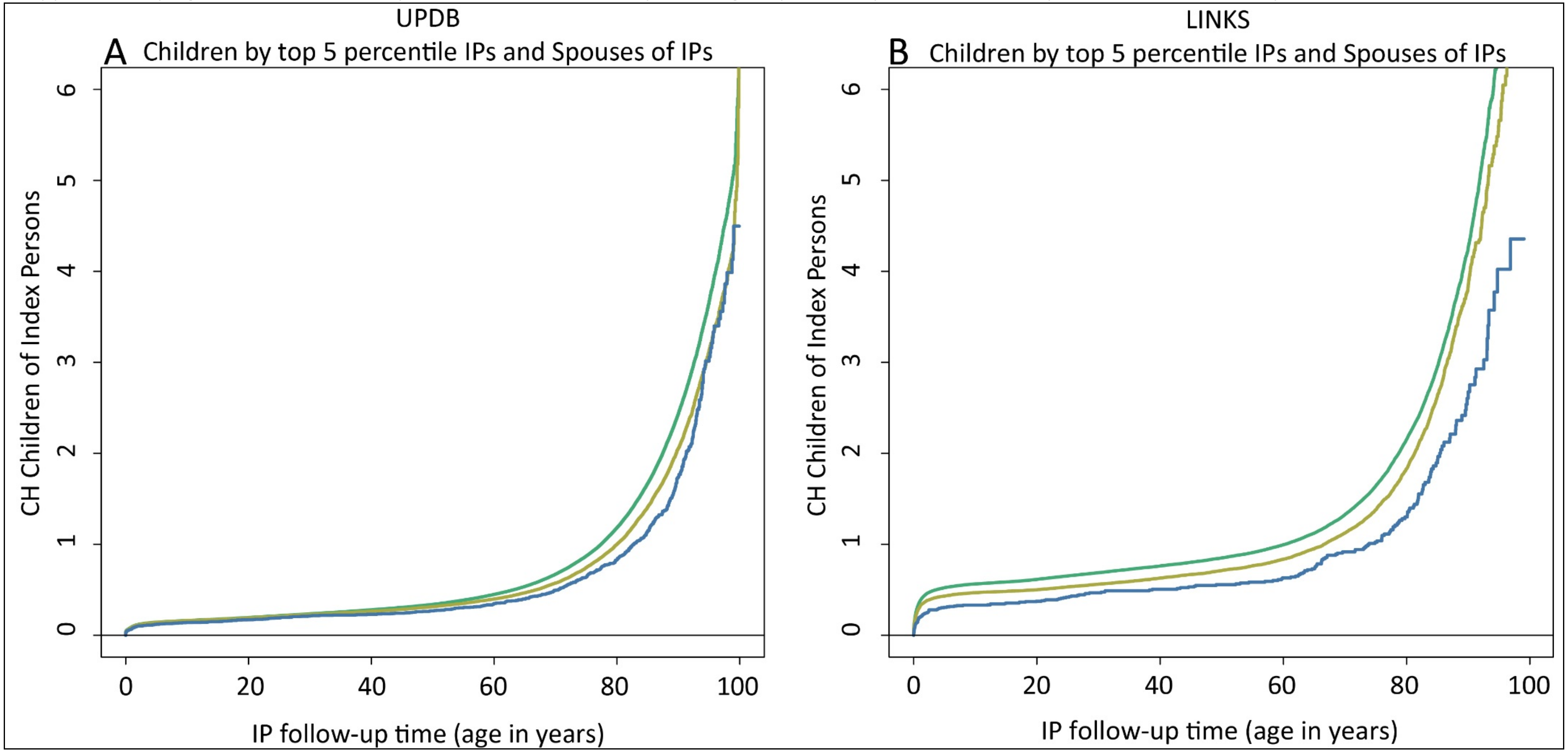

Green lines present 0 top x percentile parents or siblings, yellow lines represent 1 top x percentile parents or siblings, blue lines represent 2 or 2+ longevous parents or siblings. Panel A represents UPDB children of IPs and panel B represents LINKS children of IPs. CH=Cumulative Hazard, IP=Index Person.

Supplementary figure 6: Kaplan Meier and Nelson-Aalen plots for IPs by the longevity of their parents and siblings at the top 10%

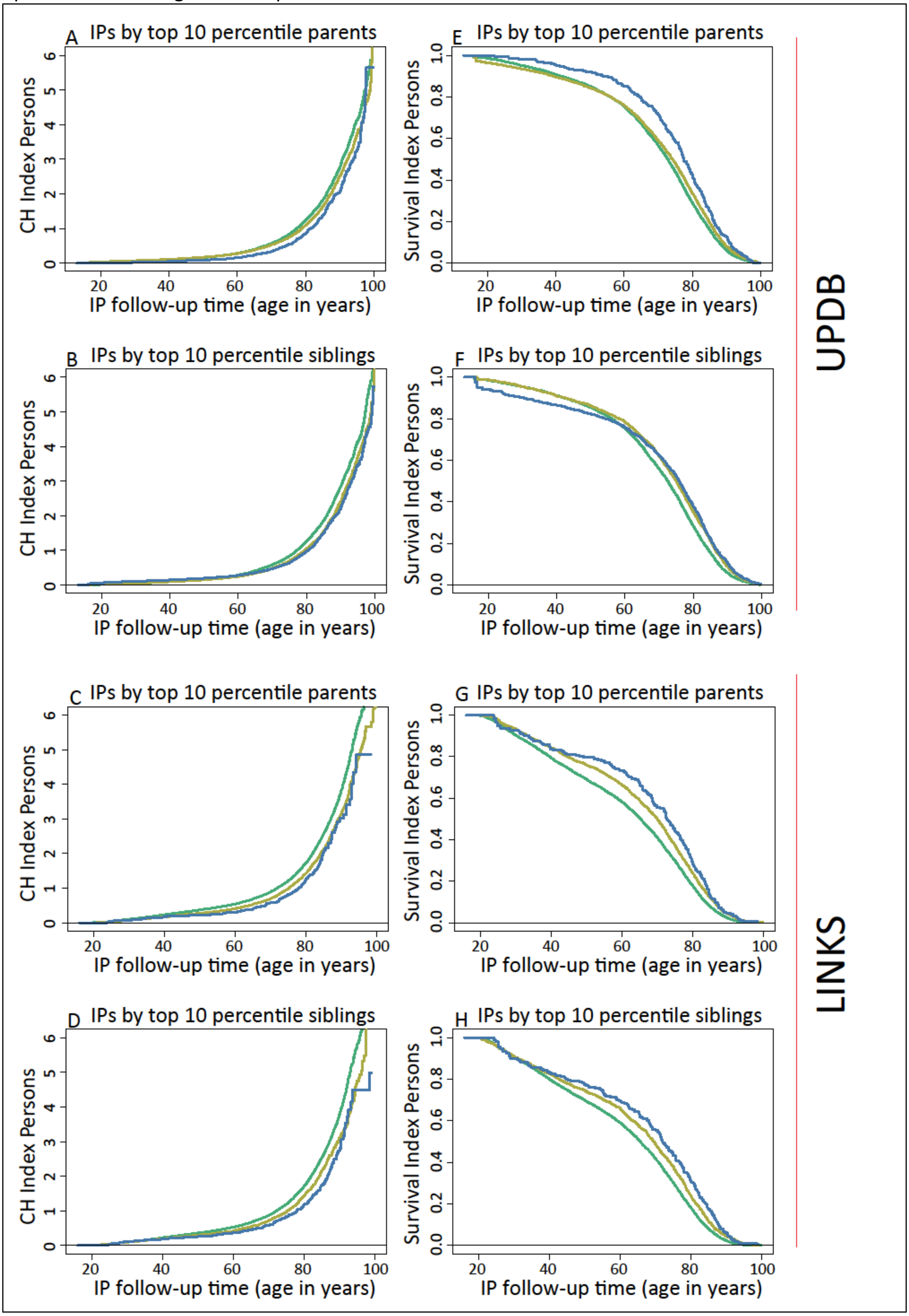

Green lines present 0 top x percentile parents or siblings, yellow lines represent 1 top x percentile parents or siblings, blue lines represent 2 or 2+ longevous parents or siblings. CH=Cumulative Hazard, IP=Index Person. Left column depicts the Kaplan-Meier curves, right column depicts the Nelson-Aalen curves. Panel A, B, E, and F represent UPDB IPs, panel C, D, G, and H represent LINKS IPs. CH=Cumulative Hazard, IP=Index Person.

Supplementary figure 7: Sex specific survival of IPs with parents belonging to the 1st until 60th percentile survivors of their birth cohort

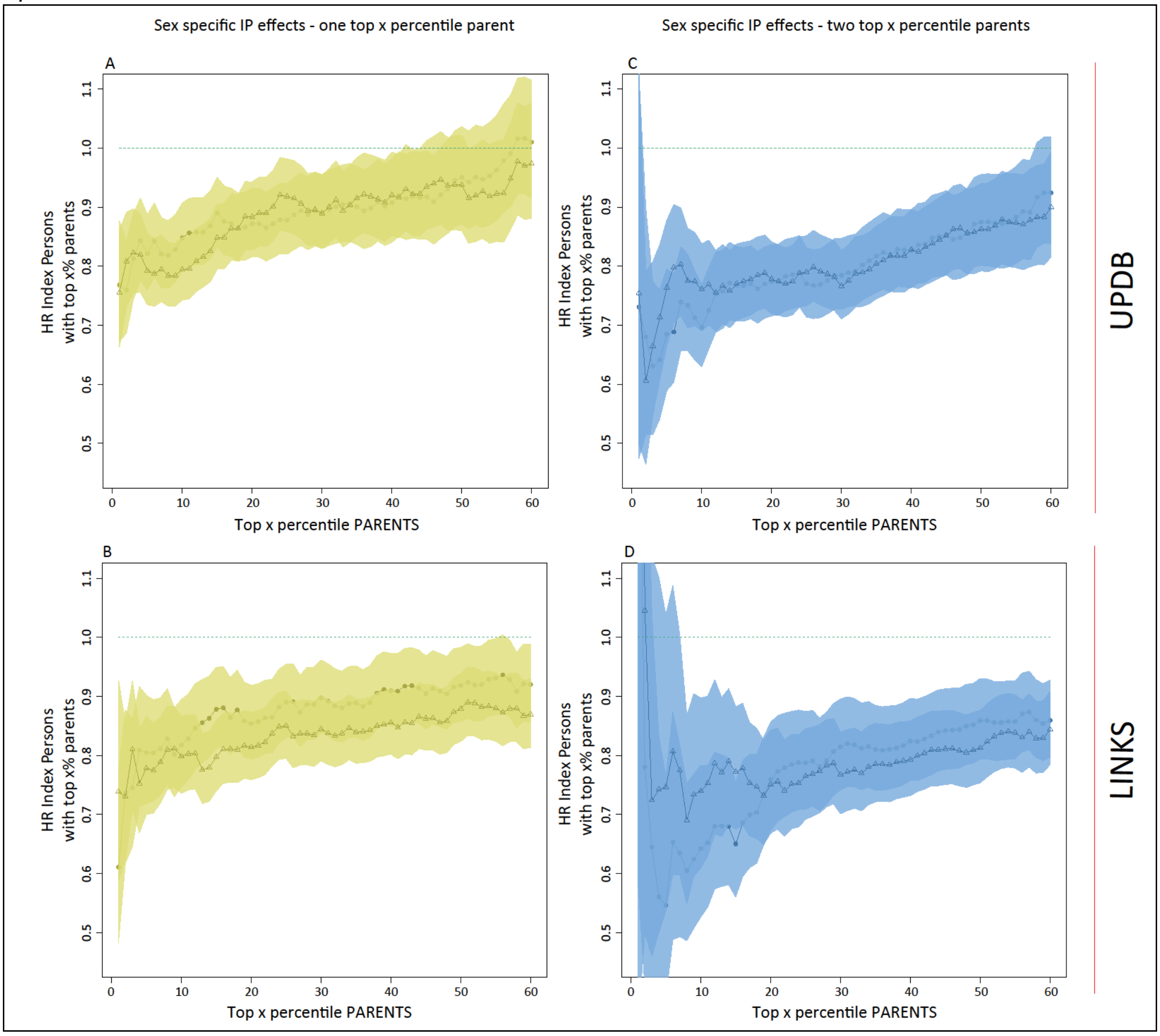

Green lines present 0 top x percentile parents, yellow lines represent 1 top x percentile parents, blue lines represent 2 or 2+ longevous parents. Round nodes=males, triangle nodes=females. Top row (panel A and C) represent UPDB IPs, bottom row (panel B and D) represents LINKS IPs. HR=Hazard Ratio, IP=Index Person. Error bars represent confidence intervals.

Supplementary figure 8: Nelson-Aalen plots for children of IPs by the longevity of their parents (IPs and spouses of IPs) at the top 10%

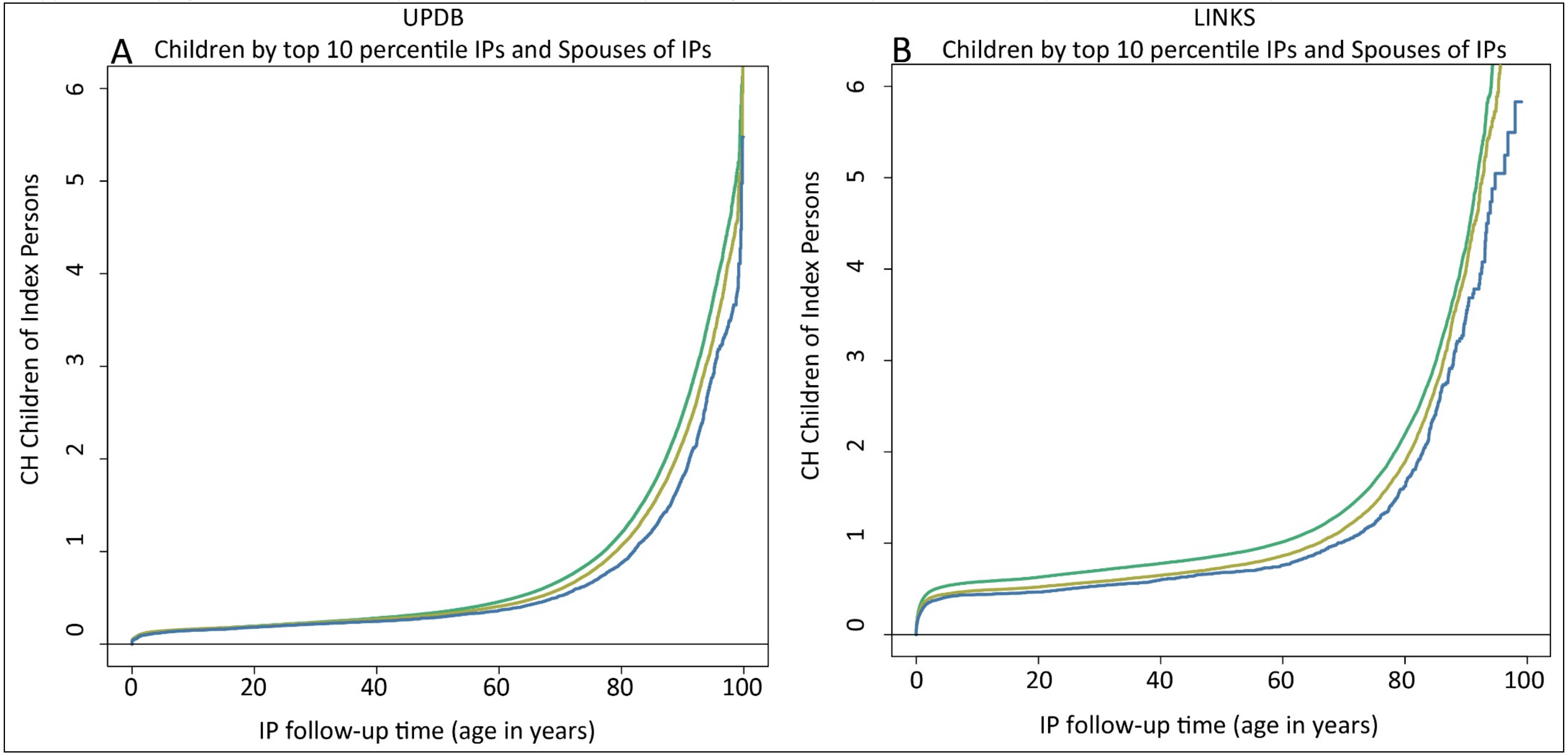

Green lines present 0 top x percentile parents or siblings, yellow lines represent 1 top x percentile parents or siblings, blue lines represent 2 or 2+ longevous parents or siblings. Panel A represents UPDB children of IPs and panel B represents LINKS children of IPs. CH=Cumulative Hazard, IP=Index Person.

Supplementary figure 9: Results represented by main figure 2 and 3 with the IP numbers split in half

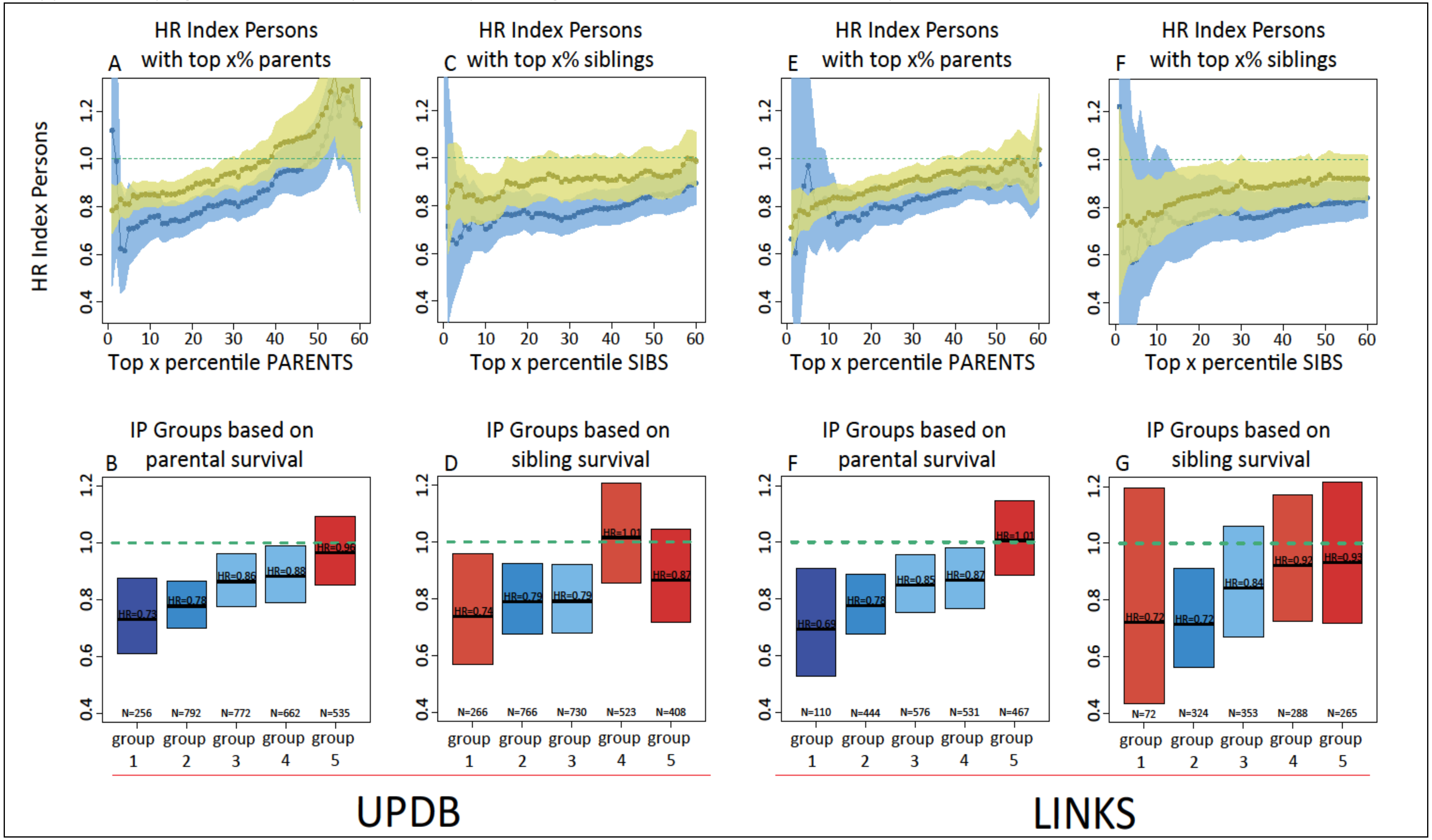

Figure illustrates results similar to main figure 2 and 3 with the number of IPs cut in half. Top row: green lines present 0 top x percentile parents or siblings, yellow lines represent 1 top x percentile parents or siblings, blue lines represent 2 or 2+ longevous parents or siblings. Bottom row: green lines represent the reference category, which is group 6. Top column (Panel A, C, D, and E) represents figure 2, bottom column (Panel B, D, F, and G) represents figure 3. HR=Hazard Ratio, IP=Index Person. Error bars represent confidence intervals.

Supplementary figure 10: UPDB and LINKS birth cohorts mapping of age by top 1, 5, and 10th percentile

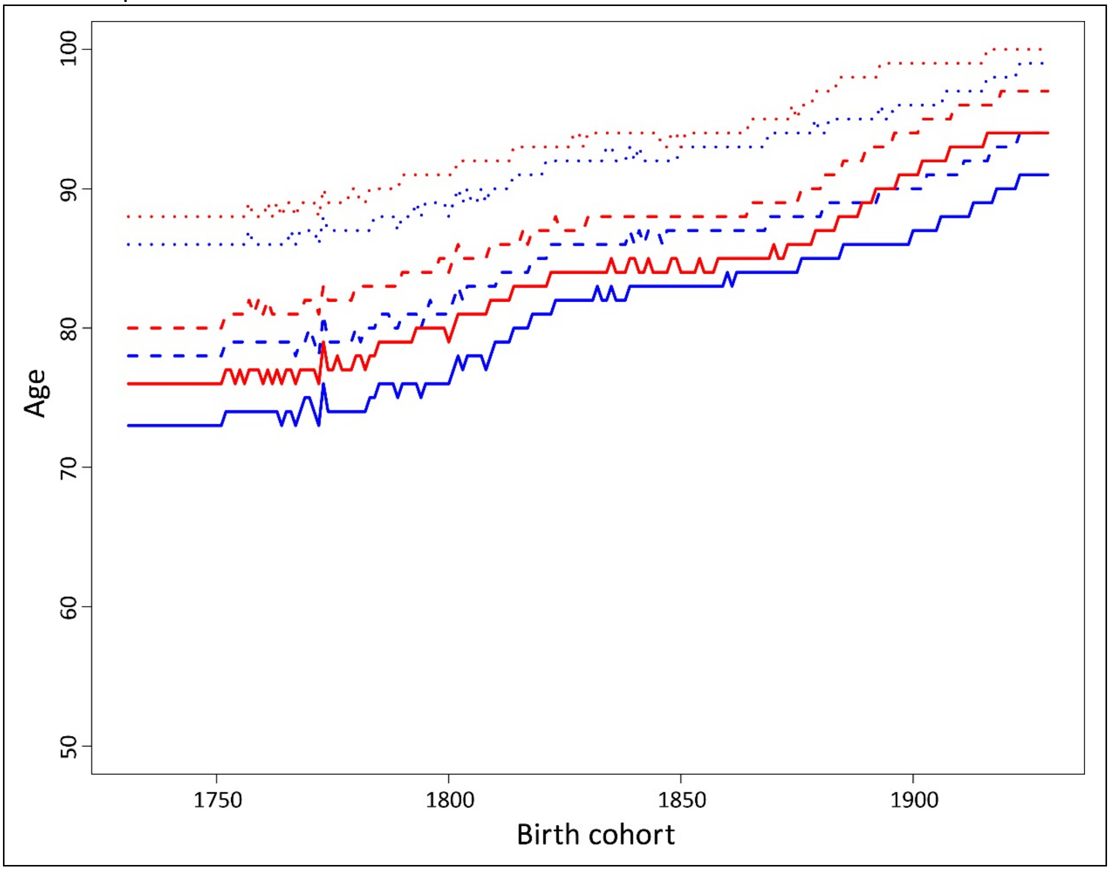

This figure represents the the percentile-age pairings from the Swedish lifetables used to calculate survival percentiles in both the UPDB and LINKS datasets. Line colors: Blue: men, Red: women. Line patterns: Dotted lines represent the top 1% survivors of the specific birth cohorts. Broken lines represent the top 5% survivors of the specific birth cohorts. Unbroken lines represent the top 10% survivors of the specific birth cohorts.
